# Comparison Between Effects of Home Based Cardiac Rehabilitation Programs Versus Usual Care on the Patients’ Health Related Quality of Life After Coronary Artery Bypass Graft

**DOI:** 10.5539/gjhs.v8n4p196

**Published:** 2015-08-18

**Authors:** Mohsen Salavati, Gholamhossein Falahinia, Ali Esmaeili Vardanjani, Hossein Rafiei, Saeid Moosavi, Mehdi Torkamani

**Affiliations:** 1 Department of Medical Surgery, School of Nursing and Midwifery, Hamadan University of Medical Sciences, Hamadan, Iran; 2 Chronic Disease (Home Care) Research Center, School of Nursing and Midwifery, Hamadan University of Medical Sciences, Hamadan, Iran; 3 Department of Medical Surgery, School of Nursing and Midwifery, Qazvin University of Medical Sciences, Qazvin, Iran; 4 Tabriz University of Medical Sciences, Tabriz, Iran

**Keywords:** cardiovascular disease, chronic disease, home based rehabilitation program, health related quality of life, developing country

## Abstract

**Background & Aim:**

To compare home-based cardiac rehabilitation with usual care on the patients’ Health Related Quality of Life (HRQoL) after coronary artery bypass graft in patients with coronary artery bypass graft (CABG) surgery.

**Methods:**

In a randomized controlled clinical conducted from March 2013 to June 2013, 110 patients with CABG surgery were randomly assigned into two groups. While patients in group I, were received usual care and patients in group II, in addition to the usual care were received home-based cardiac rehabilitation programs. The 27-item MacNew Heart Disease HRQoL questionnaire was used to evaluate the patient’s HRQoL under and over 2 months after intervention.

**Results:**

At the time of 0, mean score of HRQoL was 67.86±7.5 and 64.76±8.4 in patients in group I and group II, respectively (P> 0.05). Although mean score of HRQoL in all patients in both groups increased two month after intervention, but this increase in patients in group II were higher than patients in group I (154.93±4.6 vs 134.20±8.2). This difference were statistically significant (P< 0.05).

**Conclusion:**

Quality of Life (QoL) can be considered as a quality indicator of health care systems. Results of present study showed that home-based cardiac rehabilitation program improved patients HRQoL after CABG surgery.

## 1. Introduction

Cardio Vascular Disease (CVD) currently accounts for nearly half of non communicable diseases (Laslett et al., 2012). This disease is a leading cause of mortality, morbidity, and disability world widely (Poortaghi et al., 2013; Ebrahimi et al., 2011). The World Health Organization (WHO) estimates there will be about 20 million CVD deaths in 2015, accounting for 30 percent of all deaths worldwide (WHO, 2014). Although CVD death rates are declining in most high income countries, trends are increasing in most low and middle income countries such as our country Iran (Institute of Medicine 2010; Mehralian et al., 2014; Hatmi et al., 2007). In a review study in this regards in 2011, Ebrahimi et al., reported that prevalence of CAD among Iranian population is higher than Western countries and similar to some Middle East countries (Ebrahimi et al., 2011).

As a chronic disease, CVD affect patients’ health Related Quality of Life (HRQoL) negatively. In one study in this regards, Unsar et al., compared HRQoL in patients with and without coronary artery disease. They reported that HRQoL of patients with coronary artery disease is lower in the domains of mobility, hearing, breathing, elimination, usual activities, mental function, discomfort and symptoms, vitality, sexual activity, and total score in compared to patients without this disease (Unsar et al., 2007). Coronary Artery Bypass Grafting (CABG) is one choice for treatment for CVDs. This surgery not only improves patient’s cardiovascular status, also can improves their HRQoL. In one study in this regards, Ballan & Lee examined HRQoL in patients with CABG before and after surgery. Results of Ballan & Lee study revealed that patients HRQoL improved in domains of physical functioning, general health perception and energy/vitality after surgery significantly (Ballan & Lee, 2007).

Cardiac rehabilitation programs are an effective strategy to decrease complications in patients with CABG surgery and leading to improvement their HRQoL after surgery. Results of one study in this regards in 2013, revealed that participation in cardiac rehabilitation programs decreased ten year mortality following CABG surgery significantly (Pack et al., 2013). Although participation in centre-based cardiac rehabilitation programs is recommended to patients with CABG surgery, but one of the main problems with regards to this type of rehabilitation programs is the low participation rate (Oerkild et al., 2012). To resolve this problem, there has been an increasing focus on home-based cardiac rehabilitation program. In one study in 2012, Oerkild et al., compared home-based cardiac rehabilitation program with usual care in elderly patients with coronary artery disease in Denmark. Results of Oerkild et al., study revealed that home-based cardiac rehabilitation programs improved exercise capacity among this group of elderly patients (Oerkild et al., 2012).

Although use of home-based cardiac rehabilitation programs showed good results in improving patients outcome, but studies that examined effect of home-based cardiac rehabilitation programs on HRQoL among patients with CABG surgery is limited to few study. The aim of present study was to compare the effect of home-based cardiac rehabilitation programs with usual care on the HRQoL in patients with CABG surgery.

## 2. Methods

This study is a randomized controlled trial conducted from March 2013 to June 2013 in one hospital in Hamadan, South of Iran. The study has been obtained permission from the Ethics Board of the Hamadan University of Medical Science. Inclusion criteria were: patients with CABG surgery that have at least 2 month recovery after their surgery, ejection fraction more than 50%, negative exercise tolerance test, systolic blood pressure less than 160 mmHg, diastolic blood pressure less than 105 mmHg, age from 30 to 70 years and no motion disability. Each patient was asked to fill in a written consent form.

Eligible patients, whom were found at the time of Cardiac Intensive Care Unit (ICU) admission, were randomly assigned to group “I” or group “II” by the supervisor of the CICU, who chose the next serially numbered sealed opaque envelope containing a simple 1:1 randomization sequence. Patients in group “I”, received usual care. Usual education provided by CICU nurses in the time of hospital discharge (one hour before patients discharge, nurses visited patients in their room and answered the questions of the patients and their family) and patients in group “II”, in addition to the usual education received home-based cardiac rehabilitation programs. Home-based cardiac rehabilitation programs includes: information about their disease, usual signs and symptoms and potential complications of their illness, prescribed medications, potential change in their lifestyle (smoking cessation, dietary counseling, blood pressure and weight control, stress management), exercise programs (education and obligation walking), special signs and symptoms which they have to know in order to go to the hospital on time and any other information about the illness which patients may request to be answered. Patients in group II also received one simplified booklet about their illness. Patients and their family were carefully instructed in the training program (four sessions a week in the hospital (and 3 days left, at home based on the training given at hospital) for 5 weeks and totally 20 sessions) and guided to optimal training effort. In between the visits (three home visit includes: days 7, 27 and 47 after discharge), a telephone call was made by the nurses to resolve any questions. Patients and their families were encouraged to make contact in the event of problems to their condition. HRQoL of patients was measured by the Iranian version of the MacNew Heart Disease HRQoL questionnaire at admission time in cardiac rehabilitation center and two months after intervention. The instrument consists of 27 items which fall into three domains: 13-item assessed physical limitations domain, 14-item assessed emotional function domain, and 13-item assessed social function domain. There are 5 items that inquire about symptoms: angina/chest pain, shortness of breath, fatigue, dizziness, and aching legs. The maximum possible score in any domain is 7 (high HRQoL) and the minimum is 1 (poor HRQoL) (Asadi-Lari et al., 2013; Höfer et al., 2014). Validity and reliability of Iranian version of this questionnaire were determined in good level in previous study (internal consistency for all, emotional domain, physical and social domains were 0.95, 0.92, 0.92 and 0.94 respectively) (Asadi-Lari et al., 2013). Patient’s demographics information measured with using self designed demographics check list.

With using SPSS (Statistical Package for the Social Sciences, version 16) data were analyzed. A P-value of less than 0.05 was considered as statistically significant. Descriptive statistics (expressed as mean and standard deviation (SD), paired T-test and independent T- test for comparing the means of normally distributed independent-samples were used.

## 3. Results

**Figure 1:**
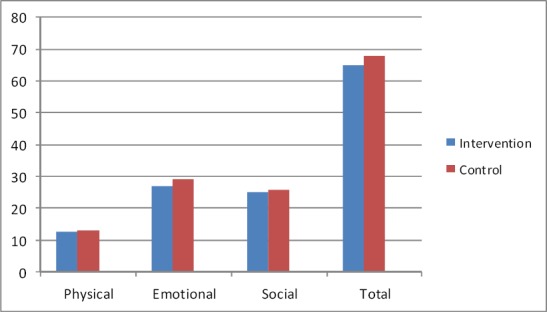
Mean score of HRQoL before program in patients in control and intervention groups

**Figure 2:**
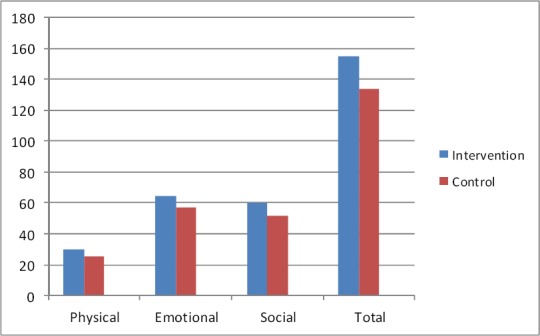
Mean score of HRQoL after program in patients in control and intervention groups

**Figure 3:**
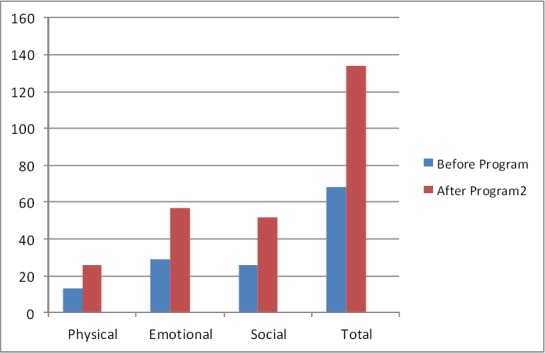
Mean score of HRQoL in patients in control group before and after program

**Figure 4:**
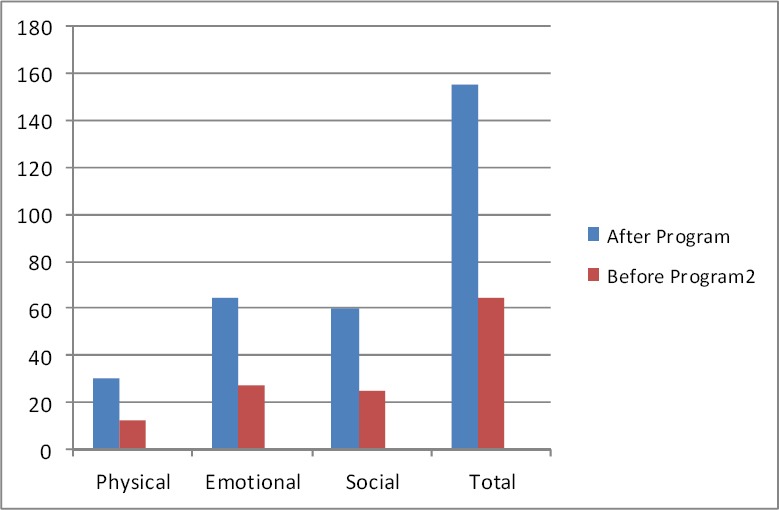
Mean score of HRQoL in patients in intervention group before and after program

Patients’ demographics information was similar in both groups (Table 1). The mean score of HRQoL in patients in group “I” and “II” before program were 67.86±7.5 and 64.76±8.4 respectively. This difference between groups were not statistically significant (P>0.05). Also mean score of HRQoL in all three sub scales were similar between groups before program. Figure 1 showed this (Figure 1). After program mean score of HRQoL in patients in groups “I” and “II” reached to 134.20±8.2 and 154.93±4.6 respectively. This difference between groups was statistically significant (P>0.05). Also this difference observed in all three sub scales (P>0.05) Figure 2 showed this (Figure 2). Paired t test were used for comparing mean score of HRQoL before and after intervention in each groups. Results of this test in patients in group “I” showed that mean score of HRQoL improved significantly after program (P= 0.001). Results of this test also showed similar findings in this regards in patients in group “II” (P=0.001). After intervention all three sub scales among patients in both groups improved significantly (P=0.001) (Figures 3, 4).

**Table 1:** Patients demographics characteristics in both groups

Item		Group “I”	Group “II”	*P value*
Age		5.25 ± 2.47	5.25 ± 2.47	*P=0.07*
Sex	Men	10	5	*P=0.136*
	Women	20	25	
	fair	1	2	
Economic status	moderate	28	26	*P=0.353*
	good	1	1	
	Primary school	24	17	
Education level	High school	3	7	*P=0.31*
	Graduate	3	5	
	Post graduate	0	1	

## 4. Discussion

Improvement in HRQoL of patients with cardiac disease is an important aims for individuals participating in cardiac rehabilitation programs (Saeidi et al., 2013). In our country Iran, similar to most developing countries centre-based programs are often the only cardiac rehabilitation program that is available (Oerkild et al., 2012) and home based programs usually isn’t available. Results of present study revealed that home based rehabilitation program improved HRQoL of patients with CABG surgery significantly.

Results of present study also showed that CABG surgery improved patients HRQoL in all domains. Similar to finding of present study, previous studies in this regards showed that patients HRQoL improved after CABG surgery. In one study in Finland, Rantanen et al., surveyed patients HRQoL after CABG surgery. They reported that patients HRQoL improved significantly after surgery (Rantanen et al., 2009). In another study in this regards, Lindquist et al., compared HRQoL of men and women patients one year after CABG surgery. They reported that both men and women patients improved in physical, social, and emotional functioning after CABG, and recovery over time is similar in both men and women. They also reported that women’s HRQoL scores remained less favorable than men’s through one year after CABG surgery (Lindquist et al., 2003). In another study Caine et al., examined patients HRQoL before and after CABG surgery. Similar to our finding, results of Caine et al., study showed that patients HRQoL improved in general health state, symptoms, and activity at three months and one year after CABG surgery. They results also revealed that factors such as reduction in waiting times for surgery, rehabilitation initiatives and more attention to the quality of information given to patients and their relatives are affection on patients HRQoL (Caine et al., 1991). Although patients HRQoL improved after CABG surgery, but some studies showed that in first month after CABG surgery patients HRQoL decreased. In one study in this regards, Rantanen et al., surveyed HRQoL in patients with coronary artery disease one month after CABG surgery. They reported that in the early stages of recovery, the HRQoL of CABG patients is lower in comparison to general population (Rantanen et al., 2008).

Despite the apparent benefits of cardiac rehabilitation programs in patients with cardiovascular disease, however use of these type of programs remain limited (Stephens, 2009). The main aim of present study was to compare the effect of home-based cardiac rehabilitation programs with usual care on the HRQoL in patients with CABG surgery. Results of present study revealed that use of home-based cardiac rehabilitation programs improved patients HRQoL significantly after CABG surgery. Limited studies in this regards showed similar finding to results of present study. In a systematic review study in 2012, Shepherd and While surveyed effect of cardiac rehabilitation program on coronary heart disease patients QoL. They reported that use of cardiac rehabilitation program improves the QoL of this group of patients significantly. They also reported that the QoL benefits of home-based cardiac rehabilitation are not inferior to those of centre-based programs (Shepherd & While, 2012). In another study in this regards, Poortaghi et al., examined the effect of home-based cardiac rehabilitation program on self efficacy of patients with cardiac disease. Results of their study revealed that home-based cardiac rehabilitation has a positive effect on patients’ self-efficacy (Poortaghi et al., 2013). Increase in patients HRQoL after home based cardiac rehabilitation could be related to patients’ nutrition improvement, physical activity improvement, risk factor reduction, lifestyle changes, and increase psychosocial well-being after implementation of this program (Hatmi et al., 2007; Lindquist et al., 2003).

## 5. Conclusion

HRQoL can be considered as a quality indicator of health care systems. Nowadays HRQoL is an important topic in treatment and caring of patients with chronic disease such as CVD. The physical restrictions and advanced symptoms derived from this disease might result in decreasing the HRQoL in patients with CABG surgery. According to finding of present study, home-based cardiac rehabilitation program improved patients HRQoL after CABG surgery. Larger clinical trials needed in this area needed to confirm our findings.
